# AMPK down-regulating Beclin-1 in prostate cancer patients with bone metastasis: An observational study

**DOI:** 10.1097/MD.0000000000041024

**Published:** 2024-12-20

**Authors:** Jiaxing Chen, Lingyun Hu, Wangguang Zhou, Chaoyang Wang, Xuewu Zhou

**Affiliations:** a Department of Urology, The People’s Hospital of Jiangshan, Quzhou, Zhejiang Province, China.

**Keywords:** AMPK, autophagy, Beclin-1, bone metastasis, molecular mechanisms, prostate cancer, signaling pathways

## Abstract

Bone metastasis is frequently seen in patients, particularly those with prostate cancer, showing a higher hazard that deteriorates the quality of life of patients, leading to poor prognosis, which eventually causes significant mortality in prostate cancer patients. The present study investigated the mechanism of prostate cancer with bone metastasis by utilizing prostate specimens from patients. A total of 418 patients were initially enrolled for clinical analysis, including age, prostate-specific antigen (PSA) levels, body mass index (BMI), prostate magnetic resonance imaging (MRI), and bone MRI, while pathological analysis included grade group and carcinoma of the prostate. Patients were divided into a prostate cancer with bone metastasis group (group 1, prostate cancer patients) and benign prostate patient group (group 2, control group) and underwent subsequent immunohistochemical (IHC) detection. Expression of AMPK/Beclin-1 signaling pathways was analyzed through immunohistochemistry. Finally, 46 patients with prostate cancer bone metastasis (prostate cancer patients) and 61 patients with benign prostate (control group) met the inclusion criteria. We examined the expression levels of Beclin-1 and AMPK in human prostate tissues by IHC and found that Beclin-1 levels were negatively correlated with AMPK in prostate cancer with bone metastasis (*P* < .05). The results of this study suggest that AMPK-Beclin-1 significantly reduces prostate cancer metastasis to the bone in human tissues.

## 1. Introduction

The most common malignant tumors in urology are carcinomas of the prostate, kidneys, and bladder.^[1]^ Prostate carcinoma involves the formation of malignant cells in the male reproductive prostate gland.^[2]^ Prostate gland development is androgen-dependent. Androgens stimulate the growth of prostate cancer (PCa), and treatments such as chemical or surgical castration and antiandrogens, which prevent androgens from binding to the androgen receptor (AR), are employed to manage the disease.^[3]^ This approach, known as androgen deprivation therapy (ADT), is effective in suppressing prostate cancer progression. Prostate cancer responsive to ADT is termed androgen-dependent prostate cancer (ADPC). However, resistance to ADT can develop, leading to androgen-independent prostate cancer (AIPC) or castration-resistant prostate cancer (CRPC).^[4]^ PCa progression involves transformation of the prostate gland structure, with the bone being the most common site of metastasis in prostate cancer, accounting for up to 90% of cases.^[5]^ The specific bone-related factors that facilitate bone metastasis in prostate cancer remain unidentified.

Bone metastasis in prostate cancer is a complex process influenced by various factors, leading to increased tumor growth and bone destruction due to altered bone stability.^[6]^ Several signaling pathways, such as the mesenchymal to epithelial transition factor, vascular endothelial growth factor, differentially methylated F13A1, NF-κB, PDGFR-α, and GDF15, have been implicated in this process.^[6–8]^ Despite its significant impact on morbidity and mortality, effective methods to inhibit the bone-forming activity in prostate cancer bone metastasis remain elusive, and the mechanisms underlying its establishment and progression are not well understood.

AMP-activated kinase (AMPK) is a key regulator of autophagy, with Beclin-1 serving as an inhibitor and AMPK as an activator.^[9]^ AMPK modulates Beclin-1 at various sites to either stimulate or inhibit autophagy. Levels of phospho-AMPK and Beclin-1 are normalized to their total levels.^[10]^ Notably, AMPK expression is significantly upregulated in prostate cancer patients.^[11]^

To our knowledge, no prior study has explored the significant up-regulation of the AMPK/Beclin-1 pathway in patients with bone metastasis of prostate cancer. This gap in research could provide crucial insights into the mechanisms underlying the establishment and expansion of prostate cancer bone metastases, aiding in early prevention and treatment strategies. While previous research has concentrated on AR in prostate cancer, the common pathway between bone metastasis and prostate cancer remains largely unexplored. Our study aims to inhibit prostate bone metastasis by examining this shared pathway. The AMPK/Beclin-1 signaling pathway is a critical common pathway found in both primary and bone metastatic cancer tissues. However, it has not been reported in prostate cancer bone metastasis patients.^[12]^ Understanding the mechanisms of the AMPK/Beclin-1 pathway and its role in regulating cellular autophagy could reduce the severity and progression of prostate cancer bone metastasis, leading to improved treatment strategies. We hypothesize that the AMPK/Beclin-1 pathway may represent a novel approach for addressing prostate cancer with bone metastasis. This study aimed to examine the expression of the AMPK/Beclin-1 pathway in prostate cancer bone metastasis.

## 2. Materials and methods

### 2.1. Patients selection

**Figure 1.**
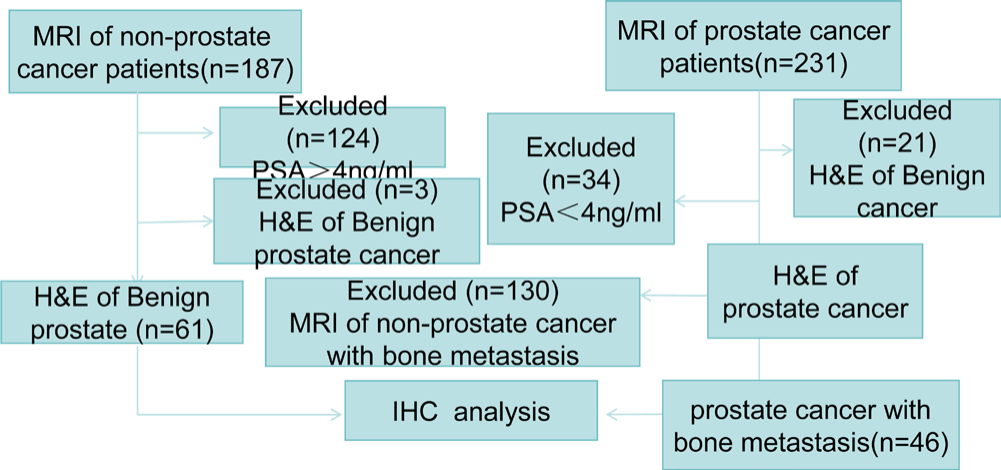
The flow chart of patient selection.

The patient selection criterion is shown in Figure 1. The common rules were to select fittest patients (Benign prostatic hyperplasia (BPH)) and PCa at random. All patients had not received prior drugs and other treatment. BPH specimens were obtained from transurethral resection of the prostate, and prostate cancer (PCa) metastases within bone tissue was obtained from prostate needle biopsies. We performed a review of data collected from the People’s Hospital of Jiangshan, Zhejiang Province, between December 2022 and September 2024. These data were collected from the patients at baseline and immediately in the context. Our study was approved by the People’s Hospital of Jiangshan, Zhejiang Province, China (NO. 2023-LW-26).

### 2.2. Materials

AMPK antibodies were obtained from Cell Signaling Technology. Anti-Beclin-1 antibody was purchased from Proteintech, and all other chemicals were purchased from Sigma-Aldrich unless otherwise indicated.

### 2.3. Methods

At the time of their clinical examination, the study was described to patients who provided written informed consent for enrollment in the follow-up study. Patients with BPH were selected according to the European Association of Urology (EAU) guidelines^[13]^ (Fig. 1). 61 BPH samples could be seen in the pathology slides matched to MRI were collected as control, and PSA were at a normal level. The material was obtained by the TURP, which was performed and the pathology revealed a BPH. The prostate tissue is a complete strip rather than comminuted tissue.

Patients with PCa metastases within the bone were selected according to the EAU guidelines.^[14]^ 46 PCa samples could be seen in the pathology slides matched to MRI were collected, and prostate-specific antigen (PSA) were at an abnormality level. The material was obtained by prostate needle biopsies, which was performed, and the pathology revealed a PCa. The needle biopsies of the prostate using a transrectal ultrasound probe during the needle insertion, the needle was inserted into the prostate from the apex to the base.

### 2.4. Hematoxylin–eosin (H&E)

Tissues were fixed in 10% formalin solution for 24h followed by embedding in paraffin. Tissue sections (3 μm) were stained with hematoxylin and eosin (H&E). The results of H&E staining were used to assess the prostate tissues. After using xylene transparent and encapsulator wet sealing, the doctor read the film and diagnosis, which reflected the degree of prostate in 5 randomly selected fields (200x).

### 2.5. Immunohistochemical (IHC) analysis

Immunohistochemical staining for Beclin-1 and AMPK was performed using rabbit anti-mouse Beclin-1 (ab10558, Abcam) and rabbit anti-mouse AMPK (ab78494, Abcam) antibodies, respectively. Briefly, sections were deparaffinized, hydrated, and subjected to heat-induced epitope retrieval using citrate buffer (pH 6.0). The sections were then incubated in 3% hydrogen peroxide solution for 25 minutes at room temperature in the dark, followed by blocking with 2% BSA in serum for 30 minutes. The sections were incubated with primary antibodies overnight at 4°C, followed by secondary antibody incubation for 50 minutes at room temperature. Immunoreactivity was visualized using 3,3’-diaminobenzidine (DAB) substrate.

Percentage of positive cells and staining intensity score: This is 1 of the most commonly used scoring methods. The positive cells were scored according to the proportion of positive cells in all tissue cells and the staining intensity of positive cells. The number of positive cells <1/3 was 1 point, 1/3 to 2/3 was 2 points, greater than or equal to 2/3 was 3 points. The staining intensity was 0 for colorless, 1 for light yellow, 2 for brown, and 3 for brown. The final score was obtained by multiplying the score of the number of positive cells by the score of staining intensity. According to the final score, the results were negative, positive or strongly positive.

Quantitative analysis of immunohistochemical staining was performed using Image-Pro Plus software (version 6.0, Media Cybernetics, Inc., Rockville, MD, USA). For each group, at least 3 fields per section were randomly selected and captured at 200x magnification under consistent imaging conditions. A uniform threshold for positive staining was set across all images. The integrated optical density (IOD) and the pixel area of the tissue (AREA) were measured, and the mean density (IOD/AREA) was calculated to represent the staining intensity. The investigator performing the quantitative analysis was blinded to the experimental groups to ensure reproducibility.

### 2.6. Statistical analysis

Data are expressed as mean ± standard deviation (SD). We used SPSS 20.0.0.0 (IBM, Armonk, NY, USA) for statistical analysis, and for evaluation and mapping, we used GraphPad Prism 8.0 software. Comparisons between 2 groups and multiple groups of measurement data were performed using 1-way analysis of variance (ANOVA) and Student *t* test. For analysis, a *P* value < .05 was considered significant for all tests.

## 3. Results

### 3.1. Patient characteristics

The clinical characteristics of the 107 patients included in the overall study are summarized in Table 1. The median age of the 2 groups was 75 years (range: 61–85 years old) in the prostate cancer groups and 71 years (range: 58–90 years old) in the BPH groups; there was no statistical difference between the 2 groups with respect to age (*P* > .05). The median PSA was 93.60 ng/mL (range: 23.90–150 ng/mL) in the prostate cancer groups and 1.26 ng/mL (range: 0.18 3.88 ng/mL) in the BPH groups, there was statistical difference between 2 groups in PSA (*P* < .05). The median body mass index (BMI) was 22.25kg/m^2^ (range:16.60–30.06 kg/m^2^) in the prostate cancer group and 23.00kg/m^2^ (range:16.90–33.20 kg/m^2^) in the BPH group. There was no statistical difference between 2 groups in BMI (*P* > .05).

**Table 1:** Clinical characteristics of patients between 2 groups.

	Benign prostate (M ± S; n = 61)	PCa with bone metastasis (M ± S; n = 46)	*P*-value
Age (yr)	72.33 ± 8.64	73.60 ± 7.04	.40
PSA (ng/mL)	1.56 ± 0.66	99.61 ± 18.61	<.0001
BMI (kg/m^2^)	23.38 ± 3.41	23.32 ± 3.78	.84

*P*-value < .05 was considered significant for tests.

Abbreviations: BMI = body mass index, PSA = prostate-specific antigen.

### 3.2. MRI findings

**Figure 2.**
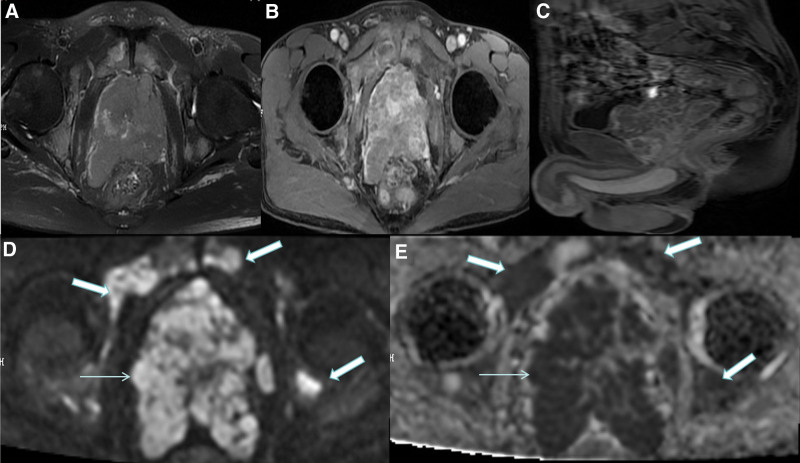
The prostate was significantly enlarged, irregular in shape, capsule destruction and adjacent structure invasion, T2WI signal of prostate was reduced, MRI showed the enhancement feature of fast forward and fast out, and multiple pelvic metastases (A–C). Prostate masses (blue arrow) and bone metastases (boxed blue arrow) were significantly limited in diffusion (D and E). MRI = magnetic resonance imaging.

**Figure 3.**
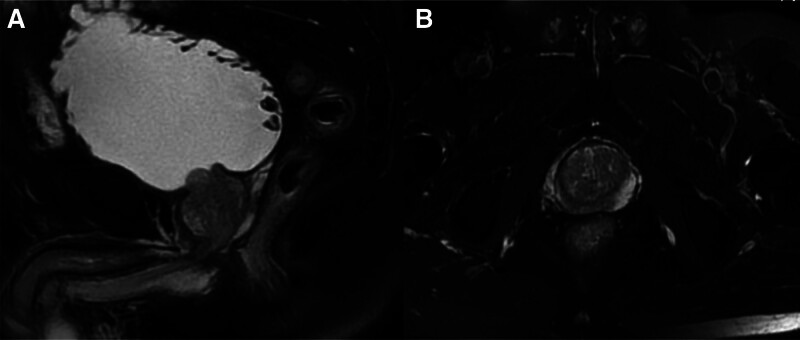
The volume of prostate increased, the boundary of prostate was clear, and the upper edge of the hyperplastic prostate protruded into the bladder (A). The prostate volume of the central zones and the transitional zones increased, the T2WI signal of prostate was moderate, mainly with stromal hyperplasia of prostate, peripheral zones of prostate was compressed and thinned, the capsule was intact (B). T2W1 = T2 weighted image MRI.

Magnetic resonance imaging (MRI) suggested infiltration into the prostate, seminal vesicles, and bones of the patients with prostate carcinoma (Fig. 2). MRI of non-prostate cancer patients suggested benign prostatic lesions (Fig. 3).

### 3.3. Histological analysis

**Figure 4.**
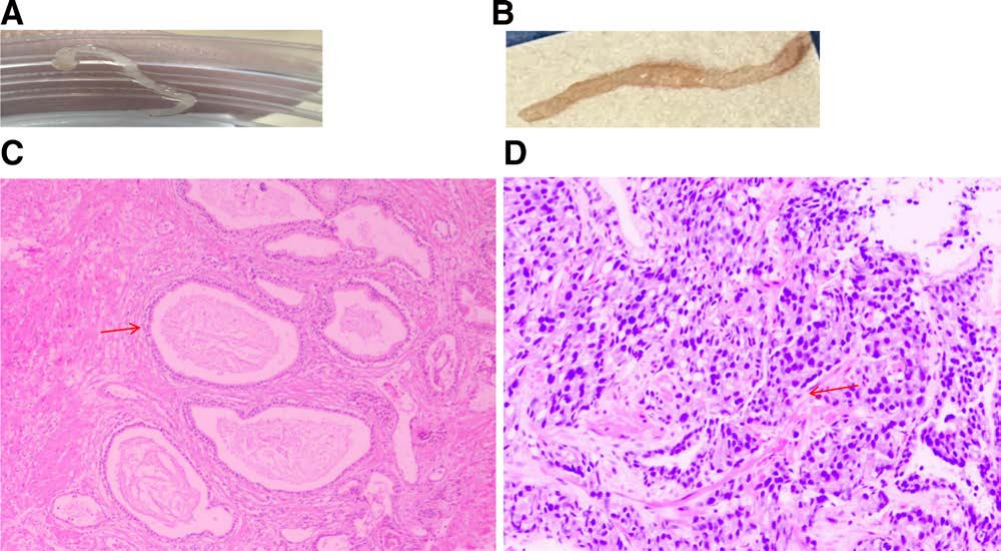
H&E, Benign prostate tissue (A, 1×), Prostate cancer puncture tissue (B, 1×), Benign prostate (C, [red arrow] normal prostate structure, large glands, and myoepithelial presence, 200×), prostate cancer puncture (D, [red arrow] increased cell density, smaller glands, nuclear atypia, solid or cribriform fusion between the glands, and disappearance of the myoepithelial structure, 200×).

Histological assessment of prostate tissue samples enabled the identification of lesions, and benign prostate tissue showed a normal prostate structure, large glands, and myoepithelial presence (Fig. 4). Prostate adenocarcinoma showed increased cell density, smaller glands, nuclear atypia, solid or cribriform fusion between the glands, and disappearance of the myoepithelial structure (Fig. 4). Benign prostate tissue (Fig. 4). Prostate cancer punctured the tissues (Fig. 4).

### 3.4. Immunohistochemical results

**Figure 5.**
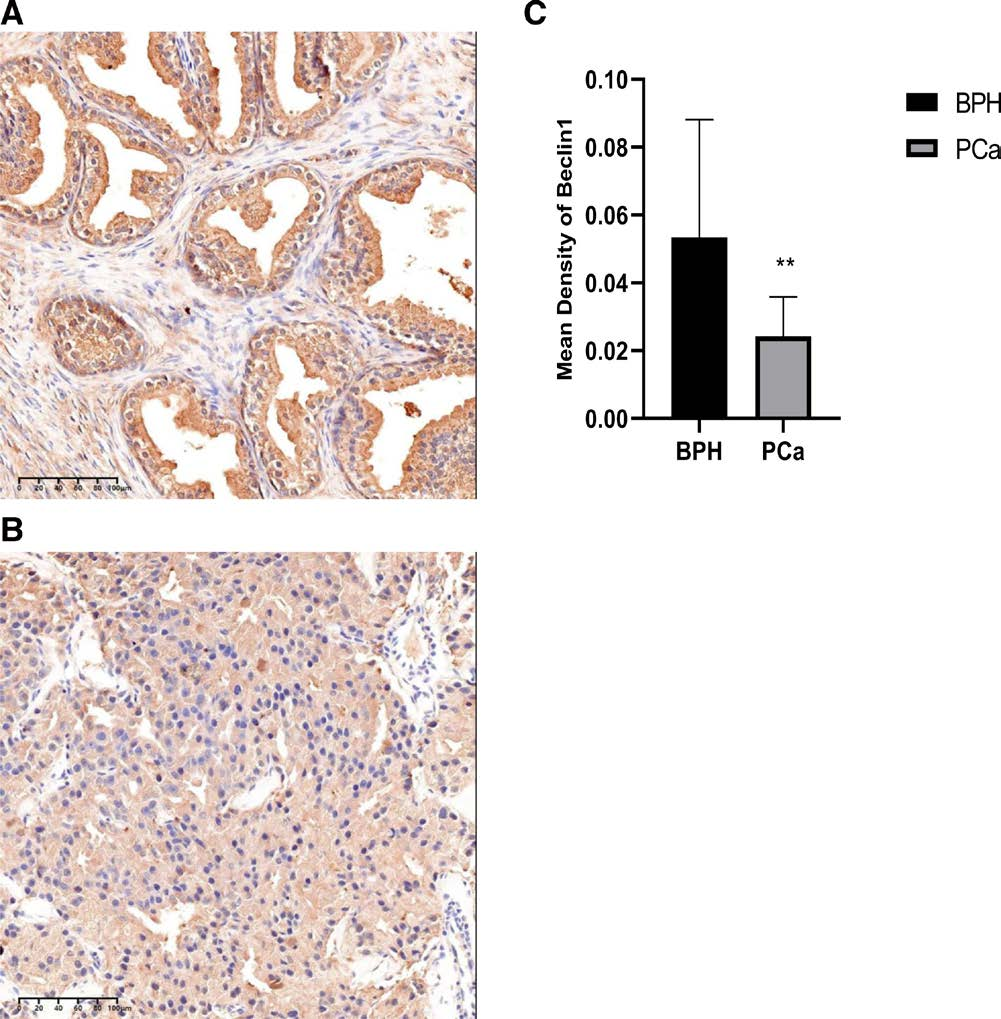
Beclin-1 expression as determined by immunohistochemistry assay. High Beclin-1 (A, scale bar 100µm) expression in benign prostate at magnification × 100; low Beclin-1 (B, scale bar 100µm) expression in prostate cancer at magnification × 100; comparison of Beclin-1 optical mean density between benign and malignant prostate (C), ** *P* < .01.

**Figure 6.**
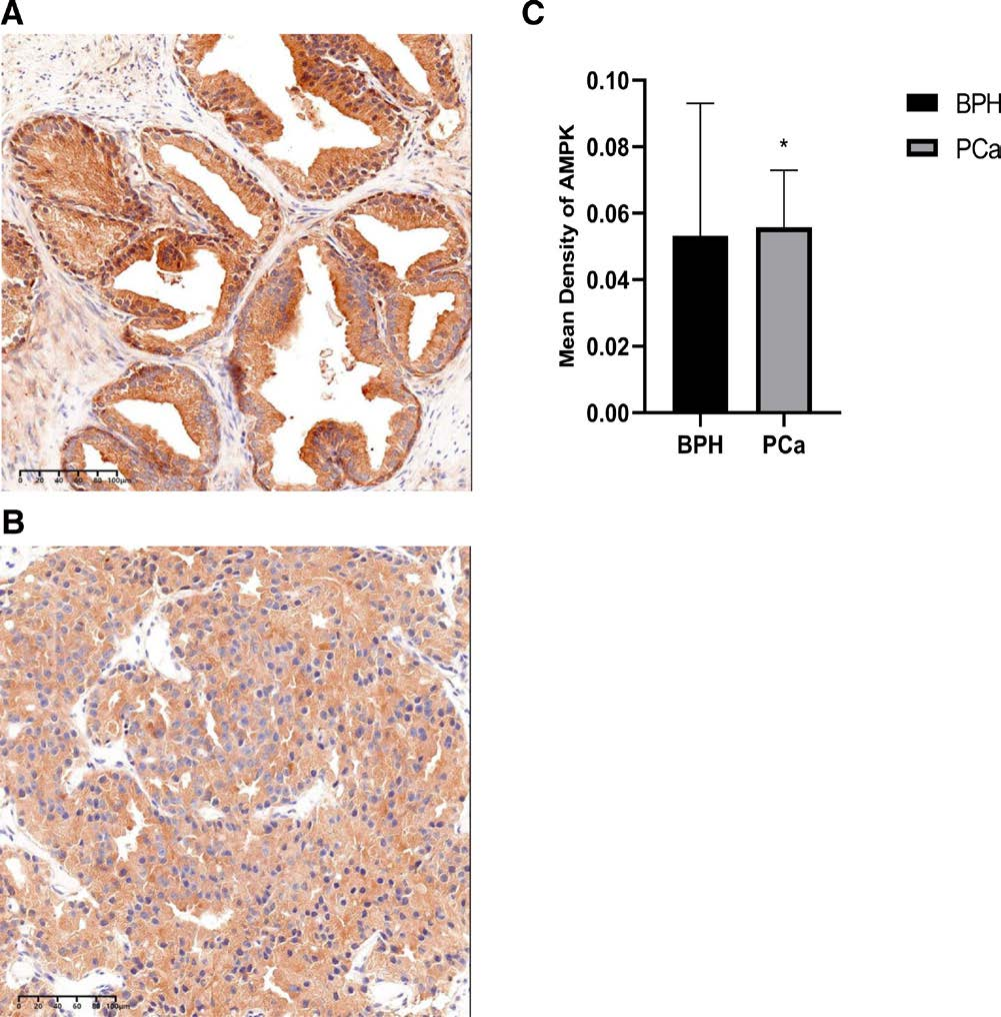
AMPK expression as determined by immunohistochemistry assay. High AMPK (B, scale bar 100µm) expression in prostate cancer at magnification × 100; Low AMPK (A, scale bar 100µm) expression in benign prostate at magnification × 100; comparison of AMPK optical mean density between benign and malignant prostate (C), * *P*<.05. AMPK = AMP-activated kinase.

Next, we examined whether Beclin-1 Expression Levels were correlated with AMPK in prostate tissues and recently discovered that the expression levels of Beclin-1 and AMPK were examined in human prostate tissues (Figs. 5 and 6). Notably, Beclin-1 levels were negatively correlated with AMPK levels in prostate cancer with bone metastasis (Figs. 5 and 6).

## 4. Discussion

Prostate cancer (PCa) is a clinically diverse disease, varying from “insignificant cancers” to “poor-prognosis cancers” that can metastasize and lead to death.^[15]^ Androgens stimulate prostate cancer growth, and treatments often involve reducing androgen activity through chemical or surgical castration or using antiandrogens to block androgen binding to the AR. Current prostate cancer treatments include surgery (such as radical prostatectomy and orchidectomy), hormonal therapy, chemotherapy, radiation therapy, radiofrequency ablation, and cancer vaccines.^[16]^ For decades, ADT has been the primary treatment for advanced prostate cancer.^[17]^ Most androgen-sensitive prostate cancer patients develop resistance to ADT within a few years, progressing to CRPC, with bone metastasis being the most prevalent form in metastatic CRPC.^[18]^ The “seed and soil” hypothesis suggests that bone metastasis relies on the interactions between tumor cells and the bone microenvironment.^[19]^ Identifying key regulators between tumor cells and the bone microenvironment can elucidate molecular mechanisms and improve clinical treatment of bone metastasis.

This study utilized prostate MRI, PSA testing, and pathological examination to identify and diagnose prostate cancer in human prostate tissue, distinguishing it from benign tissue. Distant metastases screening was conducted using computed tomography (CT) or MRI, while routine use of bone scintigraphy is not recommended. Whole-body MRI demonstrates greater sensitivity and specificity compared to bone scintigraphy in diagnosing bone metastases.^[20]^ Patients with bone metastases from prostate cancer were identified using whole-body MRI. As a control group, patients with BPH underwent the same pathological, PSA, and immunohistochemical examinations.

The resulting prostate patients were selected according to standard procedure. Hematoxylin and Eosin (H&E) stained sections were selected from representative prostate glands. Prostatic histology was evaluated with H&E for analyzing the histomorphometry. The results showed that benign prostate tissue was normal prostate structure, large glands, myoepithelial presence and prostate adenocarcinoma showed increased cell density, smaller glands, nuclear atypia, solid or cribriform fusion between glands, and disappearance of myoepithelial structure.

Depending on the cell type, AMPK activation was not automatically associated with autophagy induction. Other AMPK activators do not systematically induce autophagy and have been reported to inhibit autophagy.^[11]^ AMPK is an upstream signaling element of the Beclin-1-associated proautophagy complex, which is marked by unchecked cellular proliferation in lung tissues and potential metastasis to adjacent or distant body regions. Current results have confirmed that AMPK and Beclin-1 signaling pathways are expressed at the bone metastasis of lung adenocarcinoma.^[21]^ AMPK expression is correlated with TNM stage and distant metastasis, and Liang Wang suggested that AMPK is important in bone metastasis pathogenesis, indicating that AMPK may be an important drug target for treatment.^[22]^ Furthermore, Su et al evaluated the effects of AMPK phosphorylation on human prostate cancer cells.^[23]^ This study elucidates AMPK’s role in down-regulating Beclin-1 expression, a regulator of the autophagy pathway, in human prostate cancer with bone metastasis. The study identified distinct immunohistochemical Beclin-1 results between BPH and prostate cancer with bone metastasis. In patients with BPH, Beclin-1 expression was higher compared to those with prostate cancer and bone metastasis, whereas AMPK expression showed the opposite trend. Currently, there is no effective method to inhibit bone-forming activity induced by prostate cancer bone metastasis. This study suggests that the AMPK/Beclin-1 pathway may regulate osteoclastic activity through the autophagy pathway, promoting bone metastasis in prostate cancer cells without affecting the neuroendocrine phenotype. Research on prostate cancer with bone metastasis has primarily focused on endocrine transformation regulation via ADT.^[24]^ However, effective targets for disease progression control are scarce when castration resistance develops. The AMPK/Beclin-1 signaling pathway identified in this study may address this gap, offering a distinct mechanism for malignant cancer cells with bone metastasis that differs from previous findings. Nonetheless, the study’s results may be biased due to the lack of cell experiments, small sample size, or potential biases in patient selection.

## 5. Conclusions

In our study, AMPK-Beclin-1 significantly reduced prostate cancer bone metastasis in human tissues. Therefore, knowledge of AMPK-Beclin-1 may be useful in preventing prostate cancer metastasis. Additional research is needed to determine the primary factors linked to metastatic prostate cancer.

## Acknowledgments

We acknowledge the participants and researchers involved in this study. We thank Yao Wenlong for his interpretation of MRI and Ye Junting for pathology analysis.

## Author contributions

**Conceptualization:** Xuewu Zhou.

**Data curation:** Jiaxing Chen, Xuewu Zhou, Wangguang Zhou.

**Formal analysis:** Chaoyang Wang, Xuewu Zhou, Wangguang Zhou.

**Investigation:** Xuewu Zhou.

**Methodology:** Lingyun Hu, Chaoyang Wang, Xuewu Zhou, Wangguang Zhou.

**Project administration:** Chaoyang Wang.

**Resources:** Lingyun Hu, Xuewu Zhou.

**Supervision:** Lingyun Hu, Xuewu Zhou.

**Writing – original draft:** Jiaxing Chen.
